# D-Amino Acids and Cancer: Friends or Foes?

**DOI:** 10.3390/ijms24043274

**Published:** 2023-02-07

**Authors:** Giulia Murtas, Loredano Pollegioni

**Affiliations:** Department of Biotechnology and Life Sciences, University of Insubria, 21100 Varese, Italy

**Keywords:** amino acids, metabolism, biomarkers, cancer, NMDA receptor, proliferation, diagnostic

## Abstract

α-amino acids exist in two configurations, named D-(*dextro*) and L-(*levo*) enantiomers. L-amino acids are used in protein synthesis and play a central role in cell metabolism. The effects of the L-amino acid composition of foods and the dietary modifications of this composition on the efficacy of cancer therapies have been widely investigated in relation to the growth and reproduction of cancerous cells. However, less is known about the involvement of D-amino acids. In recent decades, D-amino acids have been identified as natural biomolecules that play interesting and specific roles as common components of the human diet. Here, we focus on recent investigations showing altered D-amino acid levels in specific cancer types and on the various roles proposed for these biomolecules related to cancer cell proliferation, cell protection during therapy, and as putative, innovative biomarkers. Notwithstanding recent progress, the relationship between the presence of D-amino acids, their nutritional value, and cancer cell proliferation and survival represents an underrated scientific issue. Few studies on human samples have been reported to date, suggesting a need for routine analysis of D-amino acid content and an evaluation of the enzymes involved in regulating their levels in clinical samples in the near future.

## 1. Introduction

Metabolic reprogramming is a peculiar trait of cancer [1,2,3]. The metabolism of L-amino acids strongly affects cancer cells [4] by: (i) establishing amino acid pools as building blocks (e.g., to generate non-essential amino acids used in protein synthesis; to produce glucose, lipids, and precursors of nitrogen-containing metabolites); (ii) epigenetic modification (e.g., producing the methyl donor S-adenosyl methionine through the methionine cycle); (iii) energetic contribution (e.g., supplying α-ketoacids for ATP production); (iv) preserving the cellular redox status (e.g., producing glutathione); and (v) detoxification of ammonia. Consequently, alterations to L-amino acid metabolism can affect various cancers in different ways.

Comprehensive reviews have summarized the effect of the amino acid composition of foods and dietary modifications on the efficacy of cancer therapies, particularly with respect to the restriction of specific amino acids to affect the growth and reproduction of cancerous cells [4,5,6,7,8]. This approach indicates a synergistic effect of diets and therapies for specific patients. For example, the inhibition of 3-phosphoglycerate dehydrogenase (PHGDH, the rate-limiting step in serine synthesis through the phosphorylated pathway) [9] is not sufficient to hinder tumor growth alone [10]; the inhibition of serine metabolism can only be achieved by both PHGDH inhibition and dietary restriction. Furthermore, as many cancers exhibit a strong need for specific amino acids from exogenous supplies or endogenous release, the selective deprivation of amino acids by the use of drugs that shut down nutrient scavenging pathways (e.g., suppressing lysosomal fusion, acidification, and nutrient export from lysosomes) has attracted great interest [11,12]. As an alternative, the pharmacological inhibition of relevant amino acid transporters may also reduce amino acid availability to cells [11,13], though targeting transporters might be a difficult task because of their broad specificity.

**Figure 1:**
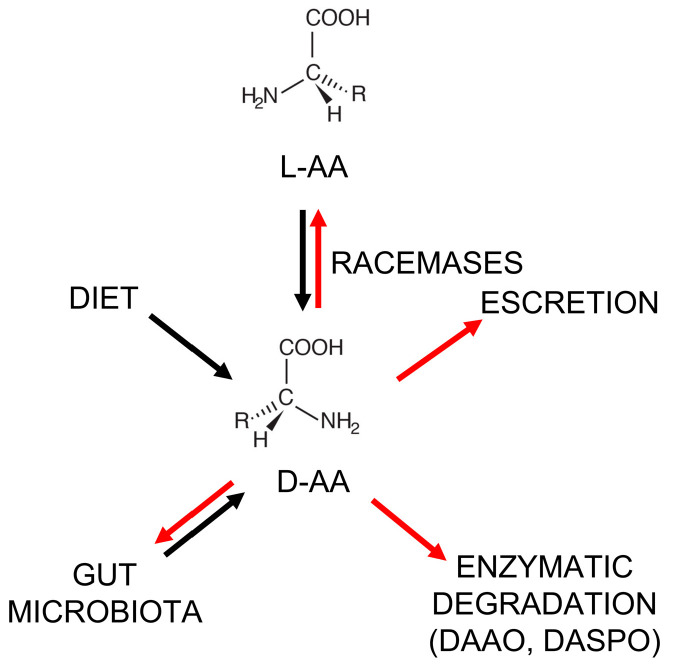
Metabolism of D-amino acids in the human body. Black arrows: synthetic pathways; red arrows: degradation/elimination pathways. L-AA = L-amino acid; DASPO = D-aspartate oxidase.

The plasma-free amino acid profile has been associated with the risk of developing cancers and, thus, could represent a promising biomarker for understanding the etiology and pathogenesis of cancers [14]. Because of the urgent need for novel potential and effective therapeutic targets and biomarkers, the D-enantiomers of amino acids (D-AA, Figure 1) have also been considered.

For a long while, D-AAs were considered non-functional and present naturally only in bacteria (as components of the peptidoglycan layer) [15,16]. However, from the 1970s, D-AAs were also identified in plants, invertebrates, and vertebrates [15,17,18,19], and then in human tissues and bodily fluids (such as the blood, cerebrospinal fluid, urine, saliva, and amniotic fluid) [20,21,22,23,24]. In animals and humans, D-Ser, D-Asp, and D-Ala represent the most abundant D-AAs in neuroendocrine and endocrine tissues. For example, in the rat frontal brain area, D-Ser is ≥ 200 nmol/g wet tissue [25]; in the rat pituitary gland, the D-Asp level is >3000 nmol/g wet tissue [26]; and in the rat pancreas, the D-Ala content reaches 450 nmol/g wet tissue [26]. In the human body, D-AAs may originate from: (i) the racemization of L-amino acids by specific racemase enzymes (but only serine racemase has been found in humans so far) [27,28,29]; (ii) diet [15,30]; (iii) microbial synthesis—the gut microbiota might strongly contribute to D-AA abundance in the human body [31,32,33] (see Figure 1). With respect to diet, the natural D-AA level in fruit and vegetables is in the 0.7–3.4% range, a figure that can increase up to 10–40% in fruit juices due to bacterial contamination. D-AAs are also naturally present in fermented foods (e.g., yogurt, cheese, kefir, soy sauce, etc.) as a result of various fermentation processes and ripening. Racemization, induced by harsh conditions, such as extreme pH values and high temperatures, is responsible for the formation of D-AAs in processed foods [15,30].

D-AAs play different physiological roles and show different activities in organisms compared to L-amino acids [16,34,35]. For example, D-Ser and D-Asp modulate the activity of the N-methyl-D-aspartate (NMDA) receptor in the brain, acting as a co-agonist and agonist, respectively (see below) [36,37]. D-AAs have been involved in pathological processes, including neurological diseases (e.g., psychosis, Alzheimer’s disease, and amyotrophic lateral sclerosis), psychiatric disorders (e.g., schizophrenia and bipolar disorder), chronic kidney disease, and cancer [38,39,40,41]. The levels of relevant D-AAs have been reported to be significantly altered in some pathological conditions compared to healthy controls [40,41,42,43,44,45] and, thus, might represent useful biomarkers (see [46]). The potential of D-AAs as therapeutic agents for treating neurological diseases and tissue/organ injury, ameliorating reproduction function, and preventing biofilm infection, has recently been reviewed [47].

With respect to cancer, D-AAs have been considered as components of tumor proteins, with the first report dated 1939 [48]. As D-Glu, as well as D-Leu, D-Lys, and D-Val, have been identified in tumor proteins, a correlation between their presence and tumor cell development was suggested. The controversial results of these pioneering studies were reviewed in 1950 [49]. Subsequently, a greater accumulation of D-AAs in tumor cells compared to the corresponding L-enantiomers was reported following the administration of ^14^C-labeled D-AAs to tumor-bearing mice [50]. However, with the advent of advanced analytical techniques in the 1980s, D-AAs were found to be absent in proteins from selected human tumors [51], and no statistically significant difference in free D-Asp and D-Glu concentrations between several tumors and healthy control tissues was observed [52]. Nonetheless, a detailed analysis of the results of the latter study showed that most tumors contained less D-Asp than the control tissues, whereas nearly half of the investigated tumors contained 1.6- to 5.4-times more D-Glu than the controls (0.72% D-Asp and 0.61% D-Glu in tumors compared to 0.94% D-Asp and 0.35% D-Glu in the control tissues). Later studies, based on advanced analytical techniques, identified significant alterations in the levels of selected free D-AAs in specific cancers (see paragraph “D-amino acids level in tumors”).

The experimental detection and quantification of D-AAs are based on four main steps: the release of the amino acids from the matrix, the separation of the individual amino acid enantiomer, detection, and quantification. The analysis of D-AAs is hampered by their small quantities and the presence of large amounts of the corresponding L-enantiomers. The reference analytical techniques are based on high-performance liquid chromatography (HPLC) and gas chromatography [53]. In recent years, alternative methods have been introduced, such as ultra-performance HPLC, super- and sub-critical fluid chromatography, and capillary electrophoresis. As a rule, these methods are expensive, time-consuming, and cannot be used in online applications. These drawbacks were overcome by the advent of selective biosensors, characterized by the simplicity and speed of operation, cost effectiveness, appropriate sensitivity, and integration into portable devices. An update of biosensors for detecting and quantifying D-AAs and their numerous applications (comprising in vivo sensors for continuous monitoring by a device implanted in the body) has been reported recently [54].

Accumulating evidence suggests that D-AAs might be involved in the pathogenesis, treatment, and detection of cancer, although human data are still limited. In the present review, we aim to summarize the relationships between D-AAs and cancer, aiming to shed light on controversial experimental data and conclusions that have accumulated over about 90 years of investigation. It is to be anticipated that many new fields of investigation and breakthroughs will appear in the future.

## 2. D-Amino Acids Level in Tumors

Several studies have highlighted alterations in D-AA levels and metabolism in the pathogenesis and progression of certain types of tumors, such as gastric cancer (GC) [55].

In 2007, for the first time, Nagata and collaborators reported a positive correlation between tumor cells and D-AAs [56]. The HPLC analysis of gastric juice samples from 18 patients with GC and 18 patients with gastric ulcers, duodenal ulcers, or chronic gastritis, carrying or not *Helicobacter pylori* infection, showed high concentrations of D-Ala and D-Pro in GC patients with *H. pylori* in comparison with the other groups, whereas the D-Ser levels were higher in the GC group not carrying *H. pylori* infection (Table 1) [56]. Interestingly, D-Pro and D-Ala were the best substrates for the action of D-AA dehydrogenase from *H. pylori* [57].

Several years later, a new, non-invasive biosensing system based on luminescent DNA/silver nanoclusters was used for the detection of D-AAs on saliva samples of GC patients (not considering the presence of *H. pylori*) and healthy subjects [58]. Consistent with the previous investigation, these analyses confirmed a 10-fold increase in the D-Ala and D-Pro contents in cancer patients compared to controls (Table 1). Moreover, a metabolomic analysis by HPLC QQQ MS/MS on urine samples from 80 healthy subjects and 84 GC patients showed that D-Ile, D-Ser, and D-Ala levels significantly increased in the GC group (the absolute values were not determined), confirming that the levels of these amino acids could be used for the prediction of GC (with a prediction accuracy of 92%) [59]. Recently, an innovative nanoenzyme-based colorimetric assay tested on saliva samples from five GC patients and five healthy subjects showed an increase in D-Ala and D-Pro levels in the GC group (Table 1) [60]. This simple, non-invasive saliva assay seems to be well-suited for the timely diagnosis of GC. Notably, P103L substitution in D-amino acid oxidase (DAAO, the peroxisomal enzyme responsible for the catabolism of neutral and basic D-AAs) [61], was identified in four female patients affected by GC (with drinking and smoking habits) as well as in one with small intestine cancer and one with colon cancer [62]. Unfortunately, the effect of this point substitution on DAAO function, stability, and subcellular targeting has not so far been investigated.

Altered D-AA levels have also been found in other tumors, such as hepatocellular carcinoma (HCC), breast cancer, and pancreatic cancer (PC) [63,64,65]. PC (accounting for 57,600 cancer cases and 47,050 cancer deaths in the United States) has the lowest 5-year overall survival rate (9–10%) of any solid tumors and a percentage of incidence and mortality which is the highest among people aged 65–74 for both sexes [66]; it is predicted that PC will represent the second-leading cause of cancer-related deaths by 2030. PC has many histologic types, beginning with neoplasms originating from ductal and non-ductal cells. The former includes pancreatic ductal adenocarcinoma (PDAC), which corresponds to 90% of total types and exemplifies typical PC (see paragraph “Proposed roles of D-amino acids in cancer”). He and collaborators analyzed serum samples from patients affected by diabetes mellitus (DM) with and without PC; LC-MS analysis highlighted that glutamine metabolism is one of the most dysregulated processes in PC [63]. A similar result was evident for HCC; LC-MS/MS analysis on serum samples of 30 healthy subjects and 30 patients revealed a significant decrease in D-Glu and D-Gln (1.9- and 1.6-fold lower, respectively, see Table 1) for HCC compared to controls, probably due to the over-expression of glutamine synthetase, an enzyme that is not enantiospecific and, thus, catalyzes the synthesis of glutamine from both L-Glu and D-Glu [64]. The levels of D-Arg, D-Ile, D-Ala, D-Met, and D-Thr were also decreased at a statistically significant level in the same patients (*p* < 0.01).

**Table 1:** D-amino acid concentration in different samples of cancer patients and healthy subjects.

D-Amino Acids	Sample	Cancer (μM)	Controls (μM)	References
*Gastric cancer*				
D-Ala	Gastric juice *	(+) 250.8 ± 79.5 (−) 26.3 ± 6.0	(+) 15.9 ± 8.5 (−) 6.6 ± 1.2	[56]
D-Pro		(+) 80.3 ± 34.2 (−) 4.0 ± 2.1	(+) 17.9 ± 11.9 (−) 12.8 ± 5.5	
D-Ser		(+) 8.5 ± 6.5 (−) 24.2 ± 10.2	(+) 3.3 ± 2.4 (−) 2.3 ± 2.0	
D-Ala	Saliva	50.6–253.2	0–25.3	[58]
D-Pro		22.5–112.6	0–11.3	
D-Ala + D-Pro		325.5–433.5	13.0–15.5	[60]
*Hepatocellular carcinoma*				
D-Ile	Serum	2323 ± 612	3475 ± 1520	[64]
D-Asp		407.3 ± 115.1	545.8 ± 254.4	
D-Ala		1092 ± 367	1521 ± 715	
D-Glu		3.29 ± 1.16	5.25 ± 2.57	
D-Met		6.47 ± 2.91	10.23 ± 5.95	
D-Gln		11.9 ± 7.2	22.2 ± 8.8	
D-Val		142.3 ± 34.4	169.1 ± 53.4	
D-Thr		85.2 ± 23.1	104.3 ± 29.4	

* (+) and (−) indicate infection or not by *H. pylori.*

Recently, metabolomic analysis of human MCF-7 breast cancer cells strongly supported the existence of significantly altered levels of certain D-AAs in comparison with MCF-10A non-tumorigenic cells. In particular, MCF-7 cancer cells contained up to 22-times more D-Asp, D-Ser, and D-Glu than non-tumorigenic MCF-10A breast epithelial cells (D-Asn, D-Ala, D-Thr, and D-Tyr levels were also higher in tumor cells) [65]. On the other hand, D-Val, D-Leu, D-Pro, D-Lys, and D-Trp concentrations were higher in MCF-10A cells. The increase in D-Asp and D-Ser levels (suggested to arise from an overexpression of some racemases or an upregulation of amino acid antiporters, see below) sustained the hypothesis that these amino acids may act as potential oncometabolites supporting cancer proliferation [65]. The authors proposed a simple index based on the relative levels of specific L- and D-AAs as a useful malignancy indicator (MI) of cancer (and as an early diagnostic marker); high MI (>60) results from increased demand for “essential” L-amino acids for cancer cell proliferation, whereas low MI (<1) arises from increased demand of specific D-AAs or the result of the release of amino acids from the cell.

## 3. Proposed Roles of D-Amino Acids in Cancer

**Figure 2:**
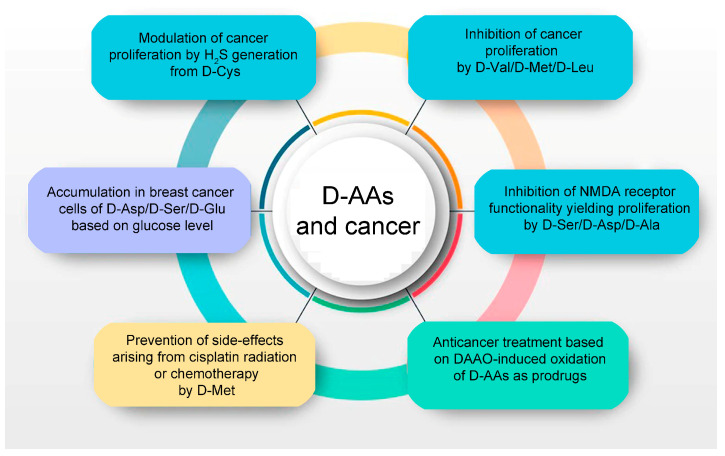
D-amino acids are related to cancer cell proliferation and metabolism (light blue and violet background, respectively), oxystress anticancer therapy (green background), and protection from side effects due to radiation or chemotherapy (orange background).

Several studies have found reduced tumor cell proliferation after the administration of D-AAs (Figure 2). D-Met prolongated the survival time of AH109A hepatoma-bearing rats (as it maintained the plasma levels of transferrin and albumin) [67] and inhibited tumor growth and protein synthesis when supplemented in the same cell line [68]. When AH109A hepatoma-bearing rats under full parenteral nutrition were treated with selected D-AAs, D-Val showed the most significant tumor growth inhibition (based on tumor volume and weight and on the resulting decreased DNA, RNA, and protein content) [69]. 

A tendency for tumor growth inhibition was also observed for D-Met and D-Leu supplementation. The D-Val application also improved the nutritional status of the host, as indicated by higher hematocrit and hemoglobin red blood cell levels. Subsequently, an inhibitory effect induced by D-Leu (at 50 mM, a very high concentration) was reported on the proliferation of MCF-7 breast cancer cells [70].

MCF-7 breast cancer cells were observed to take up several D-AAs from the extracellular medium, including D-Asp and D-Ala, whereas other D-AAs, including D-Ser, were released [65]. This evidence points to a specific uptake or release process for certain D-AAs in different cell types. The metabolism of cancer cells appears to be affected by D-AA and glucose concentrations in the extracellular environment—in the presence of a high glucose level, a net uptake of D-Ile, D-Glu, D-Phe, and D-Lys was observed in MCF-7 cells, whereas these D-AAs were released into the culture medium when the cells were cultured at normal glucose concentrations. In addition, D-Thr and D-Ser were shown to accumulate to a greater extent in MCF-7 cells cultured at high glucose conditions (Figure 2).

NMDA receptors, widely distributed in the central nervous system, are heterotetramers with subunits belonging to three subfamilies. The activation of NMDA receptors requires both the binding of glutamate and a co-agonist, first proposed as glycine [71]. D-Ser, D-Ala, and D-Asp have also been suggested as modulators (co-agonists or agonists) of these receptors [72,73,74]. D-Ser and glycine affect NMDA receptor trafficking at synapses in a subunit-dependent manner [75,76]. NMDA receptor subunits are expressed in several types of tumor tissue and in human cancer cell lines [77,78,79,80] and NMDA receptor activation plays a primary role in cell growth and viability [81]. Notably, the growth of human prostate, breast, and pancreatic cancer cells was halted when NMDA receptor activity was blocked by channel blockers, such as dizocilpine (MK-801) and memantine [81,82,83]. The same molecules inhibited the growth of human skin cells by reducing or stopping NMDA receptor activity [84]. The antiproliferative effect due to MK-801 or memantine addition was reversed by D-Ser, D-Ala, or D-Asp administration (Figure 2). A higher percentage of intracellular D-Ala and D-Asp was apparent in skin cancer cells when exposed to the NMDA receptor inhibitor MK-801. This evidence suggests a possible innovative anticancer therapy based on reducing the availability of D-Asp and D-Ser for breast cancer cells and/or acting on the D-AAs metabolic pathways, i.e., on the enzymes related to their synthesis and degradation. Intriguingly, as the growth medium did not contain D-AAs, their endogenous production implicates the presence of undiscovered synthetic systems in skin cancer cells [84].

PDAC has a strong tendency for innervation and abundant perineural invasion. It is known that neurons in PDAC express abundant neurotrophic factors, promoting the malignancy of PDAC via signaling activation [85]. The conditional axonal media contain significant levels of various amino acids, including serine and glycine (related to NMDA receptor functioning, see above), suggesting that axons release these molecules into a nutrient-poor environment. Based on the dependence on exogenous serine, human PDAC is quite heterogeneous. It has been estimated that about 40% of human PDAC cell lines depend on exogenous serine for proliferation due to the absence or low expression of two critical enzymes of the phosphorylated pathway aimed at producing L-serine, namely, PHGDH and phosphoserine aminotransferase. Notably, the repressed growth of PDAC could be rescued by a co-culture with axons in the microfluidic devices [86]: in serine-dependent PDAC under serine deprivation, reduced mitochondrial activity and a rapidly increased oxygen consumption rate were observed. Moreover, PDAC tumors with augmented levels of PHGDH show attenuated NGF, tumor innervation, and shorter overall survival. These studies highlighted the metabolic role of neurons in PDAC, pointing to the relevance of the tumor microenvironment for metabolic arrangements. Anyway, various aspects related to the relationship between serine and innervation need further investigation, mainly about the mechanisms allowing neurons to release selected amino acids. On this side, it is important to note that L-serine is mainly produced by astrocytes, whereas neurons generate D-Ser [9,29]; how the serine shuttle mechanism between neurons and glia affects PDAC metabolism is still unclear.

A further link between D-AAs and carcinogenesis concerns the relationship between D-Cys and hydrogen sulfide (H_2_S) production (see Figure 2). H_2_S has been related to the pathogenesis of several types of cancers by different mechanisms, such as activating signaling pathways involved in proliferation, migration, and invasion, enhancing angiogenesis, and stimulating cellular bioenergetics [87]. Low endogenous H_2_S production appears to promote tumor cell proliferation, whereas high H_2_S concentrations, generated from exogenous donors, may inhibit it [88]. In detail, D-Cys is metabolized by the flavoenzyme DAAO, yielding 3-mercaptopyruvate, which is further converted by 3-mercaptopyruvate sulfurtransferase to H_2_S [89]. When a mouse model of ethanol-induced gastric oxidative damage was treated with D-Cys, the gastric lesions were reduced by 90%, an effect blocked by using a DAAO inhibitor [88]. A similar process might also play a role in intestinal carcinogenesis, as H_2_S generation from D-Cys and DAAO activity was reported in the rat jejunum [90]. In view of the impact of DAAO activity on cancer cell proliferation, future studies focusing on the levels of expression and/or activity of this catabolic enzyme in different cancer types and stages are mandatory as the presence of DAAO activity was ruled out in AH109A hepatoma cells and Yoshida sarcoma cells [91].

D-Met was also reported to prevent cisplatin-induced side effects in animals without antitumor interference [92,93,94,95,96,97,98,99,100] (see Figure 2). Cisplatin is a chemotherapeutic agent widely used for the treatment of various types of cancer. This treatment frequently induces negative effects, such as ototoxicity, peripheral neuropathy, weight loss, nausea, vomiting, nephrotoxicity, seizures, hearing loss, and tinnitus. A recent exploratory phase II clinical trial reported reduced cisplatin-induced ototoxicity in humans by oral D-Met supplementation [98]. The otoprotective ability of D-Met may be based on its direct (as a free-radical scavenger) and indirect antioxidant action (by the preferential binding of cisplatin to free D-Met, by preventing efflux of glutathione from cells, and/or by protecting critical enzymes). Apart from hearing protection, D-Met protected the oral mucosa from radiation-induced cytotoxicity by providing selective defense on non-transformed human cells and decreased mucositis during chemoradiotherapy [101,102].

The enzyme DAAO has been applied in anticancer treatment strategies based on enzyme-activated prodrug therapy. In this “oxystress” approach, the injected enzyme is delivered to tumor cells where it oxidizes D-AAs (the prodrug), yielding the cytotoxic metabolite hydrogen peroxide (the active anticancer drug; for recent reviews, see [103,104]). The overproduction of reactive oxygen species (ROS) can initiate lethal chain reactions that trigger oxidative damage. On the other hand, ROS are also antitumorigenic species as they induce cellular senescence and cell death [105]. Hydrogen peroxide damages DNA, proteins, and lipids; it also crosses cellular membranes and, thus, eliminates adjacent cells in a process known as the “bystander” effect. Notably, hydrogen peroxide is cytotoxic toward both proliferating and quiescent cells: in vitro, it induces the apoptosis of tumor cells via the activation of the caspase cascade. The benefit of DAAO in the oxystress approach over other oxidases lies in the ability to regulate its activity by the administration of D-AAs (normally endogenously present at low levels) and in its peculiar biochemical properties. DAAO from the yeast *Rhodotorula gracilis* was selected based on its high turnover number, tight interaction with the cofactor FAD, stable dimeric state, and easy recombinant production [106,107,108]. Furthermore, the deep investigation of its structure–function relationships [109,110,111] allowed to generate engineered protein variants with increased activity and marked cytotoxic effects at low oxygen concentrations [112,113]. In detail, the Q144R and S19G/S120P/Q144R/K321M/A345V variants showed a significantly lower Km for oxygen (from 1.9 to 0.2 mM) that allowed the efficient generation of hydrogen peroxide even at 2% oxygen saturation. Furthermore, DAAO targeting to tumors was optimized using functionalized magnetic nanoparticles [114], by coupling with PEG [115], and by fusion with the F8 antibody specific for the extracellular domain A of fibronectin, a protein largely expressed in the subendothelial extracellular matrix of tumor tissues [116]. Furthermore, the pretreatment with zinc protoporphyrin, a potent heme oxygenase inhibitor, synergistically increased the oxystress effect induced by DAAO [116,117]. In mice bearing sarcoma S-180 tumors, PEG-ZnPP accumulated in solid tumors up to 48 h after intravenous injection and significantly reduced the required amount of DAAO [117].

## 4. Conclusions

Cancer is a devasting disease attracting storming scientific attention aimed at identifying novel successful therapies and precocious biomarkers based on the elucidation of the peculiarities in cancer metabolism. Studies from the past decades highlighted the role of L-amino acids in cancer metabolism in both a tumorigenic and tumor-suppressive way. The inhibition of amino acid metabolism is attracting increasing interest in the field of cancer metabolism and reached significant outcomes for cancer medications in vitro while requiring further optimization for its in vivo use. On the contrary, the relevance of D-AAs is still not adequately appreciated. The tumorigenic and tumor-suppressive effects observed seem to depend on the identity of the D-AA and the cancer type. Neutral D-AAs, such as D-Ala and D-Pro (but also D-Ser and D-Ile), were present at significantly higher levels in bodily fluids (e.g., gastric juice, saliva, and urine) of patients with GC [56,58,59], whereas D-Glu and D-Gln were lower in the serum of patients with HCC [64]. The extracellular level of each D-AA probably reflects an alteration in the amino acid membrane transport and/or the metabolism of tumor cells [65]. D-Met and D-Leu seem to play a protective effect by inhibiting tumor cell proliferation [67,68,69,70], especially in hepatoma and breast cancer cells. D-Asp and D-Ser, however, have been proposed as putative oncometabolites supporting cancer proliferation [65]. Notably, the latter D-AAs are modulators of NMDA receptor activity and reverse the antiproliferative effect of MK-801 and memantine channel blockers on different human cancer cells [81,82,83]. The role of D-Ala and D-Pro requires further investigation. Finally, the protective effect of D-Cys appears to be related to the inhibition of tumor cell proliferation as a result of H_2_S generated from its catabolism [88,90]. 

Because of the increased exposure to D-AAs resulting from the high current consumption of processed foods [30], it is likely that D-AA-dependent processes will become exceedingly dysregulated, and this might affect the development of cancer. Furthermore, as bacteria contribute significantly to the body’s D-AA pool, the alteration of the gut microbiota composition may also have important consequences. In view of this, more research focusing on the impact of D-AA metabolism on cancer is expected in the near future. This implies the need for routine analysis of D-AA content (strictly adhering to the good practice guidelines described in [118]) and the evaluation of enzymes that regulate D-AA levels in clinical samples.
